# Interactions between serum FSH, inhibin B and antral follicle count in the decline of serum AMH during the menstrual cycle in late reproductive age

**DOI:** 10.1002/edm2.172

**Published:** 2020-08-08

**Authors:** David M. Robertson, Chel Hee Lee, Angela Baerwald

**Affiliations:** ^1^ Centre for Endocrinology and Metabolism Hudson Institute of Medical Research, Clayton, Victoria Monash University Clayton Victoria Australia; ^2^ School of Women’s and Children’s Health University of New South Wales Kensington Australia; ^3^ Department of Mathematics and Statistics University of Calgary Alberta Canada; ^4^ Department of Academic Family Medicine College of Medicine University of Saskatchewan Saskatchewan Canada

**Keywords:** BMP15, cumulin, estradiol, GDF9, inhibin A, menopause, oocytes

## Abstract

**Objective:**

To investigate the hormonal interrelationships during the menstrual cycle in women of late reproductive age with suppressed serum AMH and antral follicle count (AFC).

**Methods:**

Serum hormones (AMH, FSH, LH, estradiol, progesterone, inhibin A, inhibin B), AFC (2‐10 mm) and AMH/AFC ratio (an estimate of AMH/follicle) were assessed every 2‐3 days across the menstrual cycle in 26 healthy ovulatory women aged 18‐50 years.

**Results:**

An 11‐fold fall in AMH/AFC was observed in women aged ≥45 years compared to those 18‐45 years (*P* < .001). Although women ≥45 years exhibited normal menstrual cycle patterns of serum estradiol, progesterone, LH and inhibin A, FSH was elevated (*P* < .001) and inhibin B suppressed (*P* < .001) compared to the younger group. Overall FSH was inversely correlated (*r* = .55, *P* < .05) and AMH directly correlated (*r* = .88, *P* < .01) with AFC; however, these relationships were curvilinear and more pronounced when AFC was low. Inhibin B was directly linearly correlated (*r* = .70, *P* < .01) with AFC across both high and low AMH/follicle groups.

**Conclusions:**

It is hypothesized that the marked fall in AMH/follicle in late reproductive age is attributed to the change in the hormonal interplay between the pituitary and ovary. The fall in AFC leads to a decrease in inhibin B and a concomitant increase in FSH by a recognized feedback mechanism. It is postulated the elevated FSH suppresses AMH either directly or indirectly through oocyte‐specific growth factors leading to a marked fall in AMH/follicle. We propose that pituitary‐ovarian and intra‐ovarian regulatory systems underpin the accelerated fall in AMH/follicle during the transition to menopause.

## Graphical abstract

This study attempts to explain why the late dramatic fall in serum AMH in late reproductive age is related to concomitant changes in serum FSH, inhibin B and AMH.

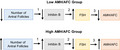

## 1 INTRODUCTION

It is recognized that the decline in ovarian reserve approaching menopause is complex, with a number of mathematical models developed to describe the decline.^1^, ^2^ Faddy^1^ described the decrease in ovarian reserve as a combination of an increase in atresia of the primordial follicle pool and a decreased progression of resting follicles into the growing follicle pool leading to an accelerated late decline. AMH has been viewed as a key contributor to this decline, as AMH is known to suppress both primordial pool atresia and follicular progression, at least in mice.^3^, ^4^, ^5^, ^6^, ^7^

The interplay between AMH, FSH, antral follicle count (AFC) and antral follicle growth dynamics is not fully understood. AMH was shown to inhibit FSH‐stimulated follicle growth, aromatase activity, LH receptor levels and progesterone synthesis in mice and humans.^8^, ^9^, ^10^ These data suggested that in the absence of AMH, follicle development and steroid hormone production were more sensitive to FSH.^5^, ^6^ Visser^10^ postulated that when AMH is decreased, the FSH‐dependent stages of folliculogenesis (ie the growth of small to large antral follicles) become more sensitive to FSH and more follicles are permitted to advance to the dominant phase. Conversely, ovarian reserve in mice was conserved by AMH administration.^11^

Both stimulatory and inhibitory effects have been reported in the regulation of AMH by FSH. In a review^7^ of human in vivo studies, serum AMH declined following exogenous FSH, and GNRH agonist/antagonist treatment.^12^, ^13^ Van Heldin^14^ investigated the changes in serum FSH and AMH in adult women following a GnRH bolus and showed a 53% increase in serum FSH within 30 minutes, followed by a 60% decrease in serum AMH by 90 minutes. This response was rapid and possibly direct without involving possible growth factor intermediaries. Roy^15^ showed a marked (>10‐fold) elevation of ovarian AMH mRNA in FSHβ^−/−^ infertile mice compared to wild‐type or FSHβ^−/−^/hFSHβ^WT^ controls, supporting the inhibitory role of FSH on AMH expression in this system. Conversely, stimulatory effects of FSH on AMH have been observed in in vitro cultures of human and monkey follicles; however, whether the FSH effect was direct or indirect through intermediaries was not established.^16^, ^17^

There is evidence that AMH is under stimulation by a range of growth factors (BMP‐2, ‐6, ‐7, ‐15, GDF9)^18^, ^19^ two of which are oocyte‐specific (BMP15 and GDF9).^20^, ^21^ BMP15 and GDF9 regulate granulosa cell proliferation and gonadotropin‐induced function probably as a heterodimer, cumulin.^22^, ^23^, ^24^ BMP15 and GDF9 have been shown to stimulate AMH both in vitro^18^, ^19^ and in vivo,^22^ although their in vitro actions on AMH are inhibited by FSH.^23^, ^24^ It has been postulated^10^ that these growth factors and AMH exert a paracrine role in regulating FSH sensitivity and thus follicle recruitment. In summary, AMH is regulated by FSH, both directly and indirectly through oocyte‐specific growth factors.

Variability in menstrual cyclicity is observed with age. While the ovarian reserve is reduced in late reproductive life, most women exhibit normal menstrual cycles with many similar attributes to those of younger women in terms of cyclicity, ovulation and steroid hormone profiles.^25^, ^26^, ^27^, ^28^, ^29^ These parameters typically change once the perimenopausal period is initiated, as reflected in oligomenorrhea, polymenorrhea, anovulation, aberrant folliculogenesis and atypical transient elevations in estradiol.^27^ In contrast, some women maintain normal cyclic behaviour up until close to menopause.^25^, ^28^, ^29^

In earlier detailed studies,^28^, ^29^ women of mid (aged 18‐35, n = 10) and late (45‐50, n = 16) reproductive age with regular ovulatory menstrual cycles were assessed every 2‐3 days across one interovulatory interval (IOI). Serum reproductive hormones, antral follicle count (AFC), antral follicular growth dynamics, luteal growth dynamics and endometrial growth dynamics were characterized across the IOI. Age‐related changes in the presence of luteal phase dominant follicles,^28^, ^29^ endometrial thickness,^30^ luteal growth^31^ and interrelationships between ovarian and pituitary hormones^32^ have been reported. The relationships between aberrant follicular dynamics, AFC and hormone production as women transition to menopause require further investigation.

It is well known^26^, ^27^ that serum AMH, inhibin B and AFC decrease in late reproductive age, as the ovarian reserve is depleted. Serum AMH is used as a clinical marker of ovarian reserve in the treatment of infertility.^12^, ^13^ AMH and inhibin B are products of the follicle. It is thought that the decrease in AMH with age was attributed to an overall decrease in the number of antral follicles. If this is the case, one would anticipate that the ratio of AMH and inhibin per follicle would remain unchanged with age. However, preliminary data from our laboratory have suggested a dramatic fall in the ratio of AMH/AFC late in reproductive life, far greater than that experienced by AMH or AFC alone.

The objective of this study was to characterize the hormonal relationships associated with the fall in AMH/AFC in late reproductive age. To this end, the interrelationships between AFC, AMH, FSH, LH, estradiol, progesterone, inhibin A and inhibin B were examined during the late reproductive age using data from a previously described study.^29^ AMH levels were expressed as serum AMH concentration and a ratio of serum AMH concentration/AFC 2‐10 mm. We tested the hypothesis that the marked fall in serum AMH with advanced reproductive age was attributed to a decrease in AMH levels/follicle and that this decrease in AMH/follicle was attributed to the action of key reproductive hormones (FSH, inhibin B).

## 2 METHODS

### 2.1 Subjects and methods

A novel set of experiments and statistical analyses were conducted using data obtained from a previous study to characterize age‐related changes in ovarian antral follicle dynamics.^29^ The study protocol was approved by the Biomedical Research Ethics Board at the University of Saskatchewan, and the Strategic Priorities and Planning Committee of the Saskatoon Health Region. Study procedures were conducted in accordance with the Tri‐Council Policy Statement on the Ethical Conduct for Research Involving Humans.

Details of subject recruitment and basis for inclusion in the study are outlined previously.^29^, ^30^ Women of mid reproductive age group (18‐35 years, n = 10) and advanced reproductive age group (45‐50 years, n = 16) were evaluated. Women were eligible if they had a history of regular ovulatory menstrual cycles (ie 21‐45 days long). Women with anovulatory cycles were excluded. Transvaginal ultrasonography was conducted as outlined previously.^29^ Scans were initiated in the early‐mid follicular phase and continued every 1‐3 days for one complete interovulatory interval (IOI). An IOI was defined as the interval from one ovulation to the subsequent ovulation. The AFC for different diameter categories (ie 2‐3, 2‐5, 2‐10, ≥3 and ≥6 mm) was quantified using serial transvaginal ultrasonography every 1‐3 days across the IOI, by a single investigator, as previously described.^28^, ^29^ AFC data for follicles 2‐5 and 2‐10 mm were examined in this study. The follicular phase was defined as the period from the first day of menses until the last day the preovulatory follicle was observed. The luteal phase was defined as the period from the day of ovulation until the day before the first day of menses. Blood samples were collected every 2‐3 days during the IOI. Serum hormone levels (FSH, LH, estradiol, progesterone, inhibin A, inhibin B, AMH (24/32, Anshlabs)) were analysed as previously described.^30^ To explore the relationship between AMH and AFC in late reproductive age, a highly sensitive AMH ELISA was devised which was able to detect AMH in 95% of samples including those in late reproductive age.^32^

Data were combined into 3‐day bins for each participant across the IOI as follows: late follicular phase 1; early, mid‐late, late and very late luteal phase; menses; and early, mid and late follicular phase (LFP1, ELP, MLP, LLP, VLLP, M, EFP, MFP, LFP2).

### 2.2 Statistics

Based on the AMH/AFC ratio data distribution with age, two groups <=45 vs >45 years were visually identified. A subsequent division into two low and high ratio groups (LRG and HRG) was statistically made, justified using a logistic regression method that maximized the separation between the two age groups. Comparison of hormone levels between stages of the menstrual cycle within the low and high AMH/AFC ratio groups was assessed by an unpaired *t* test with unequal variance and by repeated measure ANOVA (mixed‐effect model) to assess interactions (SAS 9.4). Pearson correlation coefficients were used throughout.

## 3 RESULTS

Hormone and AFC data were obtained every 2‐3 days across the IOI. The data were then averaged across the luteal phase, the follicular phase and across the IOI. AFC data used in the analyses included all detectable follicles (2‐5 and 2‐10 mm) in each phase and combined phases.

### 3.1 Changes in serum AMH, AFC and AMH/AFC with age

**Figure 1:**
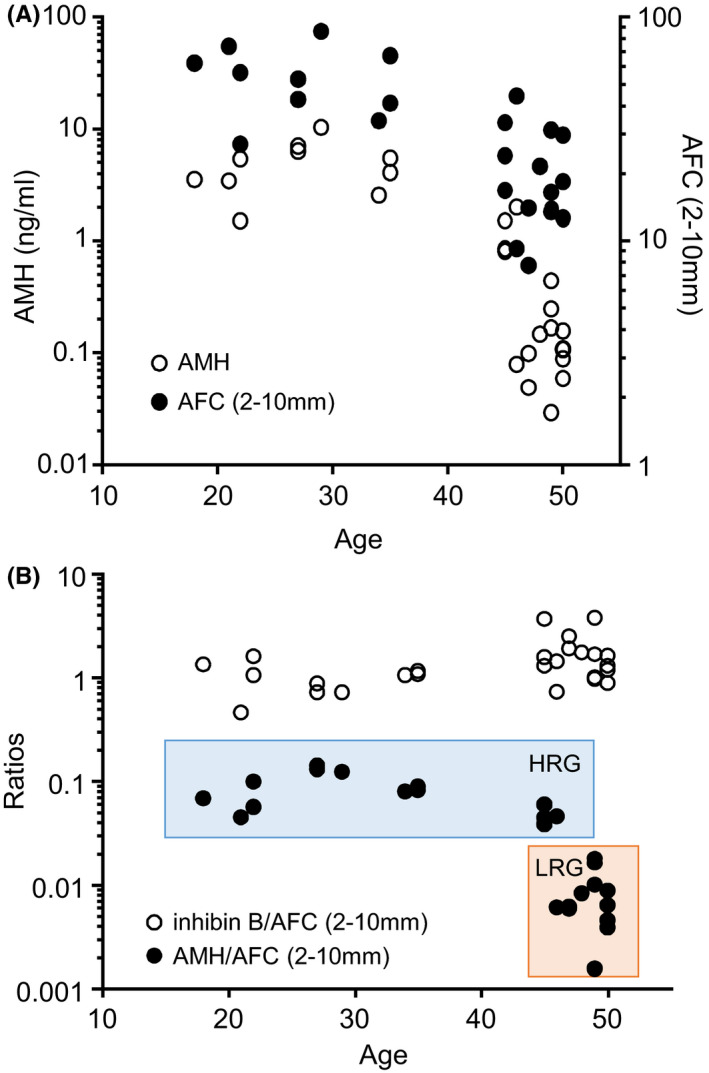
Relationship between serum AMH, inhibin B and AFC calculated as an average value across the interovulatory interval for each subject with age. Note the dramatic fall in AMH/AFC ratios (an estimate of AMH/follicle) after the age of 45 y. Panel A: AMH and AFC with age. Panel B: AMH/AFC and inhibin B/AFC ratios with age. HRG, high AMH/AFC ratio group; LRG, low AMH/AFC ratio group

Serum AMH and AFC (2‐10 mm) averaged across the IOI decreased with advancing age (Figure 1). When expressed as a ratio of AMH/AFC (an estimate of AMH/follicle), a dramatic fall was observed in 12 of 16 women in the >45 years group, reduced in individual cases to values ~1% of those in women <45 years of age. Based on this marked change in AMH/AFC ratios with age, two groups (11‐fold difference (*P* < .001, Table 1, Figure 1) were identified and designated as high AMH/AFC ratio (HRG, n = 14) and low AMH/AFC ratio (LRG, n = 12) groups. No change in inhibin B/AFC ratio was observed between HRG and LRG (Figure 1, Table 1) across the same age groups. Determination of AMH/AFC using AFC (2‐5 mm) showed similar changes to that seen with AMH/AFC (2‐10 mm) (Table 2).

**Table 1:** Demographic details, average antral follicle count and hormone levels across the interovulatory interval of ovulatory menstrual cycles in women with high and low ratios of serum AMH and AFC (expressed as ng AMH/follicle) *P < .05, **P < .01, ***P < .001. The cycle characteristics of one cycle in the LRG were incompletely described and omitted

	High AMH/AFC ratio group (HRG)	Low AMH/AFC ratio group (LRG)	*P* value	
Number of subjects	n = 14	N = 11		
Age (y)	32.2 ± 10.0	48.7 ± 1.37	.0004	***
**Cycle characteristics**				
Interovulatory interval (d)	27.8 ± 2.99	28.5 ± 8.09	.75	ns
Follicular phase length (d)	14.2 ± 2.22	14.5 ± 7.08	.90	ns
Luteal phase length (d)	13.6 ± 2.41	14.0 ± 1.83	.61	ns
Menstruation day	13.5 ± 2.47	13.9 ± 1.87	.63	ns
**Serum hormones**	n = 14	n = 12		
Serum FSH (IU/L)	5.78 ± 1.67	9.20 ± 2.04	.0001	***
Serum LH (IU/L)	9.68 ± 3.01	9.68 ± 2.56	1.00	ns
Serum estradiol (pg/mL)	71.7 ± 18.8	104 ± 51.7	.040	*
Serum progesterone (ng/mL)	5.10 ± 2.02	5.85 ± 1.89	.34	ns
Serum inhibin A (ng/mL)	31.2 ± 8.75	29.8 ± 7.31	.67	ns
Serum inhibin B (ng/mL)	48.2 ± 13.3	27.6 ± 12.3	.0004	***
Serum AMH (ng/mL)	3.99 ± 2.80	0.14 ± 0.11	.0001	***
Ratio AMH/AFC(2‐10 mm)	80.8 ± 350	8.13 ± 4.83	.0000	***
Ratio inhibin B/AFC( 2‐10 mm)	1.27 ± 0.79	1.71 ± 0.83	.18	ns
**Days to 50% rise from first ovulation**				
Serum estradiol (d)	23.1 ± 2.74	23.7 ± 6.80	.77	ns
Serum inhibin A (d)	23.8 ± 3.33	24.1 ± 6.27	.88	ns
Serum inhibin B (d)	14.1 ± 2.84	17.8 ± 6.15	.054	*
Inhibin B‐inhibin A difference (d)	9.71 ± 3.02	6.271±0.79	.003	**
Estradiol‐inhibin A difference (d)	‐0.64 ± 1.50	‐0.36 ± 1.21	.62	ns
**Antral follicle count**				
2‐3 mm	23.1 ± 9.11	11.2 ± 4.72	.0004	***
2‐5 mm	40.5 ± 16.9	15.9 ± 5.87	.0001	***
>3 mm	22.9 ± 14.3	5.96 ± 2.70	.0005	***
>5 mm	12.7 ± 8.62	3.82 ± 1.54	.002	**
2‐10 mm	46.0 ± 19.8	17.2 ± 6.01	.0001	***

**Table 2:** Antral follicle count (2‐5 and 2‐10 mm), serum AMH and AMH/follicle ratio in the follicular and luteal phases of menstrual cycles in high and low AMH/follicle ratio groups

Group	Number of subjects	Phase of Cycle	AMH (ng/mL)	AFC (2‐5 mm)	AMH (pg)/AFC (2‐5 mm)	AFC (2‐10 mm)	AMH (pg)/AFC (2‐10 mm)
High AMH/AFC ratio (HRG)	14	Luteal	3.73 ± 2.57	43.317±0.8	84.9 ± 37.4	48.0 ± 20.4	73.4 ± 33.5
		Follicular	4.22 ± 3.01	39.9 ± 18.3	104 ± 52.7	46.6 ± 21.6	86.5 ± 41.2
		*P* value	.68	.62	.55	.23	.039
Low AMH/AFC ratio (LRG)	12	Luteal	0.142 ± 0.140	15.6 ± 6.81	8.95 ± 6.23	17.2 ± 7.13	7.53 ± 5.63
		Follicular	0.120 ± 0.098	16.3 ± 5.62	8.30 ± 7.39	17.5 ± 5.65	7.10 ± 5.62
		*P* value	.52	.44	.60	.55	.21
	LP vs LP	*P* value	<.000	<.000	<.000	<.000	<.000
	FP vs FP	*P* value	<.000	.0002	<.000	<.000	<.000

Note

Mean ± SD. *P < .05.

### 3.2 Changes in AFC and hormone levels across the IOI in HRG vs LRG

No differences in the mean length of the IOI, follicular and luteal phases and day of onset of menstruation, were observed between HRG and LRG (Tables 1 and 2). In the HRG, mean AMH/AFC was 18% higher (*P* = .039) in the follicular vs luteal phase; otherwise, no difference between follicular and luteal phases within groups was detected (Table 2).

**Figure 2:**
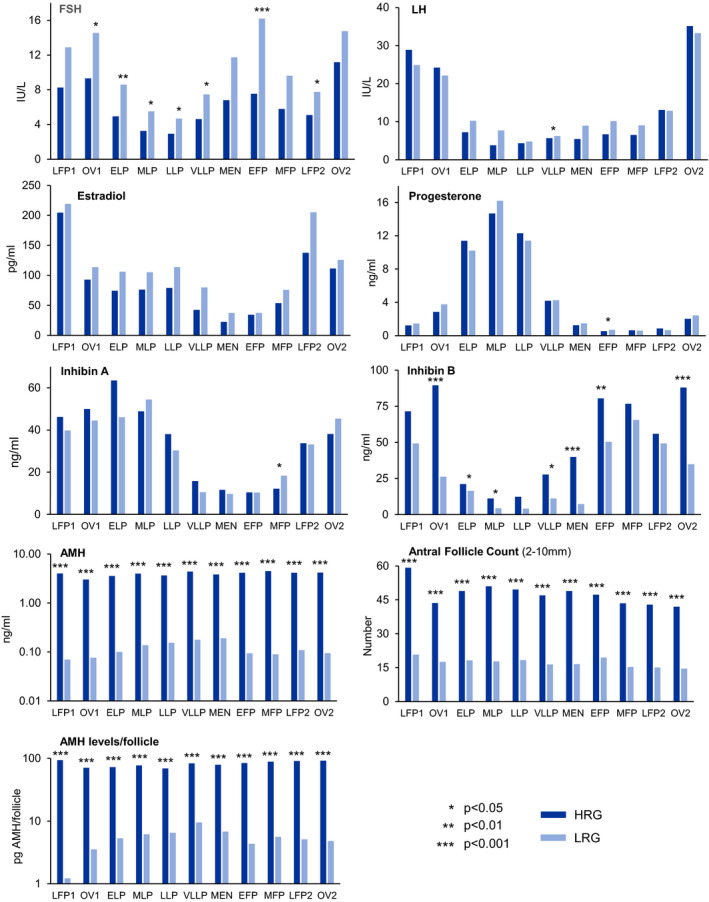
Profiles of mean serum hormones across the menstrual cycle in women that were divided into two groups based on average AMH levels/follicle (open columns, high AMH/AFC ratio (HRG); closed columns, low AMH/AFC ratio (LRG)). Serum estradiol, progesterone, AMH, antral follicle count (AFC, 2‐10 mm) and ratios of AMH/AFC and inhibin B/AFC are presented. Serum sample values have been averaged in 3‐d groups across the intra‐ovulatory interval between ovulations (OV1 and OV2) as follows: LFP1, late follicular phase, ELP, MLP, LLP, VLLP (early, mid‐late, very late luteal phase); M, menses EFP, MFP, LFP2, early, mid‐late follicular phases

Serum reproductive hormones levels were pooled into 3‐day bins across the IOI and compared between HRG and LRG (Figure 2). Patterns of serum LH, estradiol, Inhibin A and progesterone across the IOI remained unchanged between groups. However, in the LRG, differences in FSH and inhibin B were noted in the late luteal‐early follicular phase (Figure 2, Table 1). Overall, inhibin B was lower (43%) in the LRG compared to HRG while FSH was higher (59%, Table 1).

A significant interaction (*P* < .001) was observed between cycle stage and serum inhibin B between LRG and HRG but not with the other hormones or AFC. This interaction was reflected in a rise in follicular phase inhibin B that was delayed in the LRG compared to HRG (Day 18 vs Day 14 relative to OV1; *P* = .054, Table 1, Figure 2). The interval between the rise in inhibin B and inhibin A during the follicular phase was shorter in the LRG compared to the HRG group (6.3 vs 9.7 days, *P* = .01, Table 1).

### 3.3 Relationships between AFC and hormones in LRG vs HRG

**Figure 3:**
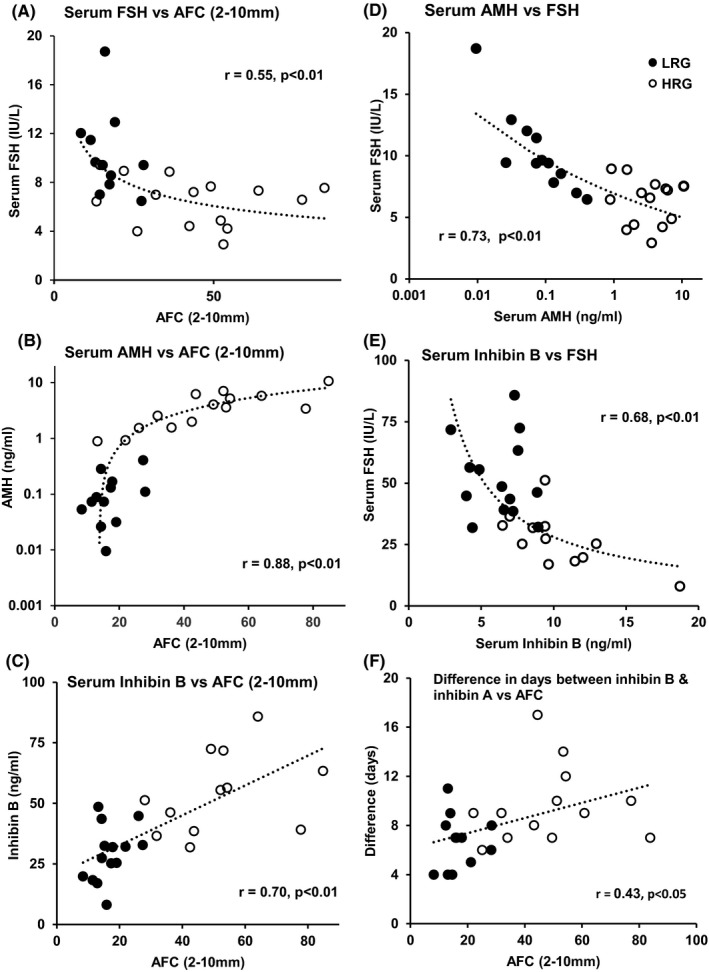
Relationship of serum AMH, inhibin B, FSH and antral follicle count (AFC) in women in two groups separated according to high or low AMH/AFC ratios (HRG (open circles) and LRG (closed circles). The data are presented as average values across the interovulatory interval for each subject. The LRG compared to HRG is characterized by significant associations between decreasing FSH (A), and increasing AMH (B) and inhibin B (C) with increasing follicle number. This is mirrored by inverse relationships between FSH and both AMH (D) and inhibin B (E). It is postulated that the increase in inhibin B with increasing AFC leads to a decrease in serum FSH by a reciprocal feedback mechanism and, in turn, an increase in AMH due to a decrease in inhibition by FSH. In panel (F), the distance in days between the rise in serum inhibin B and inhibin A across the follicular phase is plotted against antral follicle count

Regression analyses of AMH, inhibin B, FSH and AFC (2‐10 mm) between HRG and LRG (Figure 3, Table 3) revealed that the following:

1. Serum inhibin B was positively linearly correlated with AFC across both groups (*r* = .70, *P* < .01, Figure 3C).
2. Conversely, serum FSH decreased with increasing AFC (*r* = −.55, *P* < .01, Figure 3A), with the steepest decline observed in the LRG region and a flattening of the regression line in the HRG region.
3. There was an initial steep increase in AMH vs AFC in the LRG region with a flattening in the HRG region (*r* = .88, *P* < .01 Figure 3B).
4. FSH was inversely related to AMH (*r* = −.53, *P* < .01) and inhibin B (*r* = −.47, *P* < .01) with the steepest decline observed with the LRG subjects (Figure 3D,E).
5. Inhibin B and AMH were highly positively correlated (*r* = .80, *P* < .01, data not presented).
6. The interval between the rise in inhibin B and inhibin A during the follicular phase correlated with AFC (*r* = .43, *P* < .05, Figure 3F).

**Table 3:** Correlation coefficients of relationships between serum hormones and antral follicle count (AFC) presented graphically in Figure 3 and the text

Regression analysis	Correlation coefficient			
Inhibin B vs AFC	*r* = +.70	*P* < .01	Figure 3C	Figure 4 Step 1
FSH vs inhibin B	*r* = −.47	*P* < .01	Figure 3E	Figure 4 Step 2
FSH vs AMH	*r* = −.53	*P* < .01	Figure 3D	Figure 4 Step 3
AMH vs AFC	*r* = +.88	*P* < .01	Figure 3B	
FSH vs AFC	*r* = −.55	*P* < .01	Figure 3A	
Inhibin B vs AMH	*r* = +.80	*P* < .01	Data not shown	

A comparison of follicle size classes between HRG and LRG showed the expected higher AFC in the HRG (Table 1).

## 4 DISCUSSION

The availability of detailed analyses of reproductive status, hormones and AFC in asymptomatic women across the menstrual cycle with age provides us with the means to examine the underlying relationships between changes in follicle number and reproductive hormones.

In this study, we have demonstrated that although both serum AMH and AFC decrease with age, there is a dramatically exaggerated fall in AMH/AFC in older (>45 years) women. This decrease in AMH/AFC can fall to ~1% of values observed in younger women. To explore further, data were divided into two groups (high vs low AMH/AFC) and hormonal patterns and cyclicity were evaluated. Our findings show that ovulatory women in late reproductive life with low AMH levels per follicle demonstrate normal ovarian function as assessed by normal menstrual cycle characteristics, such as length of follicular and luteal phases and day of menstruation. In addition, serum LH, estradiol, inhibin A and progesterone were unchanged. Major changes, however, were noted with elevated FSH and suppressed inhibin B associated with low antral follicle count.

From these findings, we posed the following question: Is there any relationship between the marked fall in AMH/follicle with age and the change in interrelationships between FSH and inhibin B? We hypothesized that the marked fall in serum AMH in advanced reproductive age women is attributed to changes in the interplay of AFC, serum FSH and inhibin B and possibly ovarian oocyte‐specific growth factors.

**Figure 4:**
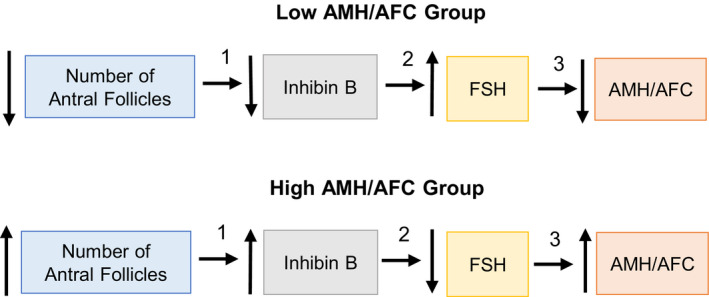
Schematic diagram of the interactions of antral follicle count (AFC), inhibin B and FSH in the regulation of AMH. In the low AMH/AFC group, the low AFC is associated with low inhibin B which in turn leads to an increase in FSH by a reciprocal feedback mechanism. The elevated FSH inhibits AMH by either a direct and/or indirect mechanism through oocyte‐specific growth factors (GDF9/BMP15). These relationships are reversed in the high AMH/AFC group. The numbered steps refer to the corresponding regression analyses and correlation coefficients presented in Figure 3 and Table 3

In Figure 4 and Table 3, we present a schematic diagram as the basis of our hypothesis, linking the changes of AMH/AFC with changes in serum FSH and inhibin B:

**Step 1. Relationship between number of antral follicles and Inhibin B:** Mean cycle inhibin B levels are directly proportional (*r* = .70, *P* < .01) to AFC across all ages (Figure 3C).
**Step 2. Relationship between Inhibin B and FSH:** At low AFC, inhibin B levels are low while serum FSH is elevated by the known feedback mechanism of inhibin B on pituitary FSH (*r *= −.55, *P* < .01) Figure 3A).
**Step 3. Relationship between FSH and AMH:** It is postulated that in the older women with low AMH/follicle, high FSH inhibits AMH either directly and/or indirectly through oocyte‐specific growth factor‐mediated regulation of AMH. GDF9/BMP15 are the most likely growth factors responsible, as they are oocyte specific and their stimulatory effects on AMH are inhibited by FSH in vitro.^16^, ^17^, ^18^, ^19^, ^20^, ^21^, ^22^ A direct inhibitory effect of FSH on AMH is also likely.^10^, ^25^
Conversely, at higher AFC, inhibin B is elevated, FSH is suppressed, and AMH levels/follicle are elevated.

In a previous study^32^ based on multiple linear regression analysis, it was concluded that the decrease in serum AMH with age in women was the major factor responsible for the age‐related increase in FSH. It was postulated that inhibin B was effective in regulating FSH within cycle but not across age, nor was estradiol thought responsible.^32^ In the present study, we attribute the age‐related increase in serum FSH to decreased AFC and thus decreased inhibin B and not to decreased AMH. The inverse relationship between AMH and FSH is thus attributed to the concomitant decrease in AMH as a result of either putative oocyte growth factor‐induced synthesis inhibited by elevated FSH or to an independent inhibitory action of FSH.

It is interesting to note that the rise in serum inhibin B in the luteal‐follicular phase transition of the menstrual cycle was delayed by an average of 4 days in women with low AMH/follicle compared to high AMH/follicle. This delay in inhibin B secretion was correlated with AFC as the largest delay was observed in women with the lowest AFC. The basis for this delayed rise in inhibin B is unclear. It is recognized that antral folliculogenesis across the normal menstrual cycle consists of a series of follicle waves, with two or three waves of follicles identified.^33^ There is evidence that inhibin B rises with the recruitment of a follicle wave at the time of selection of the dominant follicle.^34^, ^35^ It is possible that the delayed rise in inhibin B reflects an additional follicle wave preceding the ovulatory follicular phase wave.^36^ One consequence of the delay in inhibin B rise is that serum FSH is elevated for an additional period in the follicular phase, contributing, at least in part, to the overall elevated follicular phase FSH in late reproductive age.

It has been previously shown^28^, ^29^ that there is a higher incidence of cycles with luteal phase dominant follicles (LPDF) in women of advanced reproductive age compared to younger women which can lead to significantly elevated serum E2 levels in the luteal phase. In examination of the present data, 5/14 women in the HRG and 8/12 women in LRG had LPDF cycles. These data indicate that the difference in AMH/follicle between high and low AMH/follicle groups is not attributed to the presence of LPDFs, as LPDF cycles are found in both groups.

Based on the close association between AFC and serum AMH, a number of studies have explored the diagnostic use of serum AMH to forecast the onset of menopause and likely end of natural fertility.^35^, ^37^ However, based on a large meta‐analysis,^37^ the predictive value of AMH at an individual basis has been questioned. One particular problem identified in the report was that AMH is less effective in predicting menopause when assessing older women. Based on the present study, it is postulated that the marked change in AMH/follicle approaching menopause, which is in part age‐related, is a contributing factor to the reduced reliability of AMH as a marker of ovarian reserve for assessing the onset of menopause. Future studies should be conducted to evaluate the utility of AMH/AFC as a marker for predicting the onset of menopause.

In summary, based on these findings, we propose that the rapid decline in AMH in late reproductive age is attributed to a concomitant elevation of FSH, which either inhibits AMH directly or inhibits the putative oocyte growth factor‐mediated synthesis of AMH. The elevated FSH is due to a reduction in pituitary feedback by the low inhibin B levels associated with the low AFC at this stage. The reduced size of the ovarian reserve appears to be the key factor, which drives these changes. Further elaboration of this hypothesis awaits the development of suitably sensitive immunoassays for serum and follicular fluid GDF9/BMP15 to confirm their central role. Future work in this area may provide insight into the utility of quantifying AMH/follicle to provide an indication of impending loss of fertility and anticipated response to treatment in women undergoing ovarian stimulation and IVF/embryo transfer.

## CONFLICT OF INTEREST

The authors have nothing to disclose.

## AUTHOR CONTRIBUTIONS

Each author contributed equally to the study.

## Data Availability

The data that support the findings of this study are available on request from both authors. The data are not publicly available due to privacy or ethical restrictions. It should be noted that all files are permanently archived in the research laboratory of Dr Angela Baerwald, College of Medicine, University of Saskatchewan, Saskatoon, Saskatchewan, Canada.
